# The Origin and Progress of the Malignant Cholera in Manchester, Considered Chiefly in Their Bearing on the Contagiousness and the Secondary Causes of the Disease. To Which Are Added, Some Remarks on the Treatment. With an Illustrative Chart

**Published:** 1833-08

**Authors:** 


### BIBLIOGRAPHICAL NOTICE.

*The Origin and Progress of the Malignant Cholera in Manchester, considered chiefly in their Bearing on the Contagiousness and the Secondary Causes of the Disease. To which are added, some Remarks on the Treatment. With an illustrative Chart.* By Henry Gaulter, m.d., of Magdalen Hall, Oxford, &c.—8vo. pp. 206. Longman and Co., London.

Dr. Gaulter is one of the very few writers who have recently approached the much discussed, but certainly not definitely settled, subject of cholera, with sufficient ability to induce us again to recur to it: but in his work we find so very interesting a sketch of the disease as it appeared at Manchester, together with so sound and so philosophical a view of many points that have excited the attention of many, and even the temper of some physicians, that we should neither do justice to him nor to our readers, if we did not give some account of the general objects of his work.

It appears that at Manchester the local authorities did not sleep upon their posts, nor neglect their duty; for, *before* the breaking out of the disease, the town was inspected by a well-organized board of health, and, from their report, the most dreadful havoc might be fairly expected, if they were visited by the common enemy, cholera. In the quarters of the poor, there were such scenes of filth, and crowding, and dilapidation,—such habits of intemperance and low sensuality,—and, in some districts, such unmitigated want and wretchedness, that the picture of the "moral and physical state" of the poor, which was drawn by a member of the board, seemed, to the minds of many among the more easy inhabitants, as little less than a malicious libel on the town. But still, Dr. Gaulter says that the visitation of the disease was far more limited, and far less destructive, than had been predicted. "The comparative mildness of the invasion, however, far from lessening the interest of the inquiry, increases it. Can any reason be assigned why a population, represented to be sunk in the deepest poverty and immorality, should have escaped on such easy terms; why a soil, so seemingly favourable to the pestilence, should happily have yielded so scanty a produce of victims?"

Dr. Gaulter examined the first three hundred cases that occurred in Manchester. He commenced his very important investigation with a full sense of the errors and fallacies to which the inquiry was liable. The difficulty of getting satisfactory evidence from the poor and uneducated is well known: "nor are the poor the only deceivers. The inquirer is himself, from an unconscious bias, not seldom in pursuit less of truth, whatever it may be, and wherever it may lead, than of some preconceived opinion, or exclusive system. He is a contagionist, and puts but one kind of question, or hears but one class of answers: he is an adversary to contagion, and interrogates only on localities and miasm." This is most unfortunately but too true. Most men have formed some opinion before they begin their inquiries, and these are too often conducted with the *sole* object of supporting that opinion. They examine their witnesses with the same partiality that an advocate does in a court of law, whose only wish is to gain the day. If the true bearings of the case suit his purpose best, the truth, and all the truth, may probably appear; but if, on the other hand, a little *ex-parte* testimony suits his case better, why then one side of the question is very hard pushed, and the other is evaded. That such ought not to be, but that such *is* the spirit with which medical investigations are conducted, cannot be denied, and must be deeply lamented by every real philanthropist. In justice to Dr. Gaulter, we must say that he is one of the "few impartial." Judging from the mode in which his inquiries were made, and from the inferences he draws from the information he obtained, it appears certain to us that his object is truth, not victory.

At Manchester, as in many other places, the precautionary measures adopted were far from adequate to the intended purpose: Dr. G. humourously observes, that "the board resolved on doing something, seemed to have copied the example of the country gentleman described by Addison, who thought to keep out the crows by nailing up his park-gate." "But," says Dr. G., "whatever the precautionary measures had been, the same result would have followed; for the coaches did not bring it, and it neither sailed here, nor glided suddenly into the midst of us, propelled by the force of steam: it arose upon the spot." Of the correctness of this opinion he gives ample evidence. Notwithstanding the contrary impression which so generally prevails, nothing appears to Dr. Gaulter better attested in the records of this epidemic, as nothing is more remarkable, than that, while it is undoubtedly communicable from man to man, its dissemination over so large a part of the habitable globe has been, on the whole, affected without contagion, by the successive springing up, sometimes in distant, but more commonly in adjacent places, of the poison, whatever this may be, that causes the disease.

Dr. G. follows up this opinion by making some very pertinent remarks respecting the fallacious modes of argument adopted by those who assert that the disease is always, or never, contagious; and it must be regarded as a strong proof of his ingenuity and originality of thought, that he finds room to say much that is new upon a subject that we really thought had been worn threadbare, and deprived of the slightest remnant of novelty.

In the *treatment* of cholera, the one great indication, in the author's belief, is to stop the serous hemorrhage from the bowels, or to assist nature in that spontaneous effort to suppress it which she seldom fails to make. The venous injection was tried in about eight cases, all of which terminated fatally. The saline remedies of Dr. Stevens were not confided in, having been found ineffectual in the stage of collapse, and inferior to the ordinary practice in the incipient stage. The cold-water plan of Dr. Shute was adopted in two or three instances, without the extraordinary benefit which attended the use of it in his own hands. We should have been glad to know, in more precise terms, *what* benefit was obtained from this plan. It might not be "extraordinary," and still sufficiently advantageous to justify its repetition. The only novel practice which was thought to succeed was that of administering repeated doses of tartar emetic; and upon this plan of treatment there is a very interesting letter from Mr. Langford, at p. 151.

